# Opinion on weight management in the age of new technologies

**DOI:** 10.11604/pamj.2023.45.7.35023

**Published:** 2023-05-03

**Authors:** Hamid Chamlal, Rekia Belahsen

**Affiliations:** 1 Laboratory of Biotechnology, Biochemistry and Nutrition, School of Sciences, Chouaib Doukkali University, El Jadida, Morocco

**Keywords:** Weight management, new technologies, nutrition transition, overweight, diet

## Abstract

In a Moroccan context characterized by the resurgence of metabolic diseases and over nutrition, an emergence of digital media in the daily life of individuals, has led to the expansion of the use of digital diets for therapeutic or aesthetic purposes. This work raises the question of the effectiveness of unguided nutritional approaches and the associated consequences, with potential effects on the health of individuals.

## Opinion

Over the last decades, the world has been experiencing a nutritional transition characterized by changes in populations' eating habits and lifestyles. Indeed, overweight, obesity and certain chronic diet-associated diseases are in constant increase [1]. This situation has led many people to look for dietary alternatives to heal themselves, lose weight or maintain a normal body size. In order to achieve this result, the majority of people adopt different diets from different sources which are not without consequences and lead to many health problems. On the other hand, the emergence of the new technologies has facilitated and made easier to informing, communicating, socializing and performing a variety of other online activities [2]. The expansion of digital media in individuals´ daily life raises the question on the possible involvement of information in the increase of weight of digital dieters. Due to the large number of obese people, which increased from 13.2% to 20% between 2000 and 2018, Morocco seems to be a favorable ground for practices that accentuate this problem. Women would appear the most vulnerable, with 29% of them being obese and 34.4% being overweight [3].

In addition, solutions to the various bodily changes are sought by women in practicing drastic cures or by the adoption of fashionable diets, applied individually in most cases via social networks without any medical or dietary advice. These repeated cures end up failing for lack of a global approach taking into account all aspects of the person. The desire to lose weight in a limited time comes up against the body reactivity manifested by a weight stagnation and leading to shock followed by frustration due to the failure of the adopted diet, generally characterized by quantitatively limited portions, qualitatively inadequate and protein-oriented. The consequence is a deficiency in several vitamins, especially fat-soluble ones. This means that the traditional approach based on portions does not overcome the various challenges. On the other hand, a holistic approach, both medical and dietary, would make it possible to follow, accompany and monitor the case throughout the dietary treatment. It would also make it possible to always find solutions and alternatives to any encountered obstacle. A diet must comply with the principle of the Mediterranean model, balanced in terms of quantity and quality, taking into account eating habits and consumption patterns integrating the sustainable diet aspect [4]. The approach must not omit physical activity and the commitment of the patient, which constitute the essential pillars for achieving the expected objectives.

Admittedly, in the current era, the accessibility of nutritional information via the Internet is an undeniable asset. However, its use without verification of its safe origin, without advice or supervision by a competent professional, dietician or nutritionist, can lead to weight gain due to the yo-yo effect with, in some cases, serious clinical consequences. Furthermore, any nutritional information is not valid for all people and must be personalized and adapted to each case particularity. The situation reveals the problem of the appearance, on the media and social networks of quackery and the emergence of pseudo-specialists in this field advocating information and advice that can be harmful to health. On the other hand, any personalized nutritional information must come from nutritionists, framed in a nutritional project, based on the partnership of the patient and the healthcare team to achieve the desired objectives.
